# Editorial: Strategies to overcome the barriers to effective inhaled treatments

**DOI:** 10.3389/fddev.2023.1253709

**Published:** 2023-07-19

**Authors:** Philip Chi Lip Kwok, Mohammad A. M. Momin, Basanth Babu Eedara, Shing Fung Chow

**Affiliations:** ^1^ Sydney Pharmacy School, Faculty of Medicine and Health, The University of Sydney, Sydney, NSW, Australia; ^2^ Department of Pharmaceutics, Virginia Commonwealth University, Richmond, VA, United States; ^3^ Center for Translational Science, Florida International University, Port St. Lucie, FL, United States; ^4^ Department of Pharmacology and Pharmacy, Li Ka Shing Faculty of Medicine, The University of Hong Kong, Pokfulam, Hong Kong SAR, China; ^5^ Advanced Biomedical Instrumentation Centre, Hong Kong Science Park, Hong Kong, Hong Kong SAR, China

**Keywords:** pulmonary delivery, inhalation, formulation, inhaler, aerosol, flow rate, pressure drop, deposition

Inhalation administration delivers drugs into the respiratory tract for local or systemic action, but various barriers need to be overcome to ensure effective dosing and therapeutic response. Formulation, inhaler device, and patient are the three major aspects that affect pulmonary drug delivery and are important considerations as they can render aerosolisation and/or pulmonary deposition variable. Inhaled drug delivery is challenging because, unlike the more popular routes of administration (such as oral or parenteral delivery), the generation and fate of aerosols depend on many factors. For example, aerosolisation efficiency of a powder may be influenced by the formulation (e.g., particle size distribution, cohesion), inhaler device (e.g., physical design, airflow resistance, dispersion mechanism), and patient (e.g., oropharyngeal geometry, peak expiratory flow rate, pressure drop generated). Furthermore, potential interactions between multiple factors will increase the variability in aerosol performance. This Research Topic explores how problems in inhaled drug delivery may be solved to optimise treatment.

Clark’s mini review gives an overview on the advances in pulmonary drug delivery in the past 5 decades (Clark). Although metered dose and dry powder inhalers (MDIs and DPIs) have been available since the mid-1950s and early 1970s, respectively, a major spike in the development of alternative inhaled formulations and devices was triggered by the banning of ozone-depleting chlorofluorocarbon propellants in the mid-1990s. However, the pace of inventing new inhalation technology soon slowed down when environmentally friendlier propellants were developed so the MDI is still the most used pharmaceutical aerosol device by the number of doses and units sold. Much of the novel aerosol formulations and inhalers failed to be commercialised due to technical, high costs, or clinical difficulties (Clark).

Inspiratory flow rate and inspiratory pressure drop are common metrics for evaluating the performance of passive DPIs. There has been constant debate on which one of these better determines aerosol performance. The tendency to define a universal minimum flow rate for lung dosing for DPIs may be misleading (Clark). This is because the flow rate required for sufficient powder dispersion and pulmonary deposition depends on the resistance of the inhaler and the pressure drop that the patient can generate. Therefore, a one-size-fits-all threshold flow rate is not possible. Clark and Weers proposed that pressure drop is the more appropriate parameter than flow rate as it accounts for the effect of inhaler resistance (Clark; Weers).

It should be noted that drugs indicated for asthma and chronic obstructive pulmonary disease are relatively safe and have a wide therapeutic index so their prescribed doses are usually high enough to be on the upper plateau of the sigmoidal dose-response curve (Gonda; Weers). Thus, variable fine particle doses due to flow-dependence may not translate to a difference in the *in vivo* clinical response (Gonda; Weers). However, despite this relative insensitivity of therapeutic effect to the lung dose, most currently marketed DPI products have high oropharyngeal deposition. Besides being wasted and causing local adverse effects, deposition in the mouth-throat region is also a major source of intra- and inter-patient variability in the lung dose (Gonda; Weers). The opinion article by Weers and its commentary by Gonda provide food for thought for the pharmaceutical industry to leave the current comfort zone of traditional DPI technology and develop/commercialise innovative DPI products that can attain flow-independent lung doses with minimal oropharyngeal deposition. This is especially relevant for drugs with a narrow therapeutic index.

Regardless of the type of inhaler, all devices should be simple to use and ensure adherence by the patient (Gonda). Gauani et al. reported a spray dried treprostinil palmitil powder for pulmonary arterial hypertension that produced a consistent fine particle dose between 1.5–5.4 kPa pressure drop using the high resistance RS01 Model 7 DPI (equivalent to 40–80 L/min) (Gauani et al.). This inhaler is simple to use by a patient so it was chosen for this formulation. The data indicated that the aerosol performance was relatively independent of the inspiratory pressure drop or flow rate generated across that inhaler, which would minimise intra- and inter-patient dosing. A variant of the RS01 DPI was used for testing a powder containing GDC-A, a hydrophobic, crystalline drug. This powder was made by spray drying a suspension of the drug using PulmoSphere™ spray drying technology, which increased the drug loading and allowed high fine particle doses of up to 25 mg to be administered from a Size 3 capsule for this inhaler (Tarara et al.).

Dry powder inhalers may not be suitable for paediatric patients, who normally breath through the nose rather than the mouth. Transnasal pulmonary delivery using nasal cannula and prongs may be a potential solution, by using inline mesh nebulisers on a gas delivery system. The larger nebulised droplets would be trapped in the line so that smaller droplets (1.6–2.4 µm) are emitted from the nasal prongs. These small droplets can travel past the nasal cavity and deposit in the lungs. Although *in vivo* transnasal pulmonary delivery in paediatric studies are limited, there are promising data from *in vitro* lung models, computational fluid dynamic simulation, and *in vivo* animal studies. Further knowledge and experience in this mode of aerosol administration would enhance its efficiency and efficacy.

There are still plenty of challenges in inhalation drug delivery but research in this field is active and solutions are constantly being explored. The goal is to benefit patients by optimising treatment and achieve target health outcomes.

